# Clinical Outcome of Edoxaban vs. Vitamin K Antagonists in Patients with Atrial Fibrillation and Diabetes Mellitus: Results from a Multicenter, Propensity-Matched, Real-World Cohort Study

**DOI:** 10.3390/jcm9061621

**Published:** 2020-05-27

**Authors:** Vincenzo Russo, Emilio Attena, Anna Rago, Enrico Melillo, Pierpaolo Di Micco, Andrea Antonio Papa, Giovanni Napolitano, Antonio D’Onofrio, Paolo Golino, Gerardo Nigro

**Affiliations:** 1 Chair of Cardiology, Department of Medical Translational Sciences, University of Campania “Luigi Vanvitelli”—Monaldi Hospital, 80131 Naples, Italy; doc.emelillo88@gmail.com (E.M.); paolo.golino@unicampania.it (P.G.); gerardo.nigro@unicampania.it (G.N.); 2 Cardiology Unit, San Giuliano Hospital, 80014 Naples, Italy; emilio.attena@hotmail.com (E.A.); giovanni.napolitano@aslnapoli2nord.it (G.N.); 3 Department of Cardiology, Monaldi Hospital, 80131 Naples, Italy; anna_rago@alice.it (A.R.); andreantoniopapa@libero.it (A.A.P.); donofrionat1@gmail.com (A.D.); 4 Fatebenefratelli Hospital, 80123 Naples, Italy; pdimicco@libero.it

**Keywords:** direct oral anticoagulants, edoxaban, atrial fibrillation, diabetes mellitus, stroke prevention, bleeding risk

## Abstract

Diabetes mellitus (DM) is a chronic metabolic disease which is independently associated with unfavorable clinical outcomes in patients with atrial fibrillation (AF). Few real-world data are available about the clinical performance of non-vitamin K oral anticoagulants (NOACs) among patients with atrial fibrillation and diabetes. The aim of our propensity score-matched cohort study was to compare the safety and effectiveness of Edoxaban versus well-controlled vitamin K antagonists (VKAs) therapy among this population. In this study, we considered patients with AF and diabetes on Edoxaban or VKAs therapy included in the multicenter Atrial Fibrillation Research Database (NCT03760874). The occurrence of major bleedings (MB) and thromboembolic events (a composite of ischemic stroke, transient ischemic attack, systemic embolism) was respectively considered primary safety and effectiveness outcome. We identified 557 AF patients with diabetes who received Edoxaban (*n*: 230) or VKAs (*n*: 327) treatment. After propensity score matching analysis, 135 Edoxaban and 135 VKA recipients with similar clinical characteristics were evaluated. The mean follow-up was 27 ± 3 months. The incidence rate of thromboembolic events (TE) was 3.0 per 100 person-years (1.11 in Edoxaban vs. 1.9 in the VKA group, hazard ratio (HR): 0.59; 95% confidence interval (CI), 0.14 to 2.52; *p* = 0.48). The incidence rate of major bleedings (MB) was 3.7 per 100 person-years (1.2 in Edoxaban vs. 2.7 in the VKA group, HR: 0.43; 95% CI: 0.10 to 1.40; *p* = 0.14). The incidence rate of intracranial hemorrhage was 0.35 per 100 person-years in Edoxaban vs. 0.74 in the VKA group (HR: 0.49; 95% CI: 0.05 to 5.54; *p* = 0.56). A positive net clinical benefit (NCB) of Edoxaban over VKAs was found (+1.39). Insulin therapy (HR: 1.76, *p* = 0.004) and glycated hemoglobin (HR: 1.17, *p* = 0.002) were found to be independent predictors of TE; moreover, the concomitant use of antiplatelet drugs (HR: 2.41, *p* = 0.001) was an independent predictor of MB. Conclusions: Our data support the hypothesis of the safety and efficacy of Edoxaban for use in patients with AF and diabetes, justified by a favorable NCB over VKAs.

## 1. Introduction

Diabetes mellitus (DM) is a chronic metabolic disease independently associated with AF development [1,2], especially in long-lasting diabetes and in the presence of reduced glycemic control [3]. The concomitance of AF and DM leads to higher hospitalization rates, worse clinical cardiovascular mortality, and lower quality of life compared to AF patients without diabetes [4]. The unfavorable clinical course of AF patients with DM may be related to the increased risk of thromboembolic events (TE) due to high prevalence of hypertension, insulin-resistance, advanced age and vascular disease [5,6]. According to the current guidelines, a direct oral anticoagulant (DOAC) is preferred over a vitamin K antagonist (VKA) when oral anticoagulation is initiated in eligible AF patients with diabetes [7]. Recently, a randomized control trial (RCT) subanalysis showed Edoxaban, a once-daily oral direct XA inhibitor, had similar efficacy compared to warfarin, while also reducing bleeding and cardiovascular mortality, irrespective of diabetes [8]; currently, there are no real-world studies supporting these results. The aim of our propensity score-matched cohort study was to compare the safety and effectiveness of Edoxaban versus well-controlled VKAs therapy among AF patients with diabetes with atrial fibrillation in a real-world setting.

## 2. Materials and Methods

Data for this study were sourced from the prospectively maintained Atrial Fibrillation Research Database (NCT03760874) shared by three cardiologic centers in Italy (Monaldi Hospital, Naples; University of Campania “Luigi Vanvitelli”, Naples; San Giuliano Hospital, Naples) which includes all patients with non-valvular AF followed at our institution between March 2013 and July 2018. Follow-up data were obtained through outpatient visits every 3 to 6 months. During the follow-up visits, clinical status, occurrence of stroke, transient ischemic attack (TIA), systemic embolism (SE), major bleeding (MB), and clinically relevant non major bleedings (CRNMB) were assessed according to the International Society on Thrombosis and Hemostasis (ISTH) criteria [9]. All patients provided written informed consent before inclusion in the database, and the local institutional review committee approved the study. The database was queried for patients with AF and concomitant diabetes mellitus who were prescribed Edoxaban once daily or VKA. Patients with a follow-up ≤360-days from the first qualifying anticoagulant prescription or VKA patients with time in a therapeutic range <70% were excluded from the analysis. Potentially eligible Edoxaban and VKA patients were propensity score-matched to generate an analysis cohort with minimal differences in baseline characteristics. The primary safety outcome was the occurrence of MB events. The primary effectiveness outcome was thromboembolic events defined as the composite of ischemic stroke, TIA, and SE. The secondary effectiveness outcome included death from any cause; the secondary safety outcome included CRNMB events.

## 3. Statistical Analysis

The Kolmogorov-Smirnov normality test was used to analyze data normality. Continuous variables were reported using the mean and standard deviation. Categorical variables were indicated as frequency counts and percentages. Baseline characteristics between Edoxaban and VKA groups were compared by t-test for continuous variables and chi-squared test for categorical variables. The nearest neighbor propensity score matching method, without replacement and without the use of a caliper, was used to minimize the differences in baseline characteristics between patients receiving Edoxaban versus VKAs. The variables included in the propensity score were: age, sex, BMI, hypertension, CHA_2_DS_2_-VASc score, HAS-BLED score, heart failure, antiplatelet drugs, prior stroke/TIA, coronary artery disease, chronic kidney disease, left atrial diameter and indexed left atrial volume. The ratio of matching was 1:1. The incidence of thromboembolic and bleeding events was calculated both as the incidence rate every 100 patient-years and as cumulative incidence. A survival analysis was performed using the Kaplan-Meier method, and survival estimates were compared using the log-rank test. Among the matched groups, the effects of all clinically relevant baseline variables were first tested in a univariate analysis for the primary and secondary endpoints by using Cox proportional regressions analysis.

Subsequently, a multivariable model was performed including variables with a *p*-value less than 0.1 at univariate analysis. Adjusted hazard ratios (HRs) and 95% confidence intervals (CIs) were estimated for each endpoint. A two-sided *p* value less than 0.05 was considered significant for all tests. The net clinical benefit (NCB) was calculated in order to obtain an integrated assessment of the antithromboembolic and prohemorrhagic effects of Edoxaban vs. VKAs with the following formula: NCB = (thromboembolic events incidence rate with VKAs − thromboembolic events incidence rate with Edoxaban) − weighting factor × (intracranial hemorrhage incidence (ICH) rate with Edoxaban − ICH incidence rate with VKAs), where we used a weighting factor of 1.5, as previously described [10]. All statistical analyses were performed using STATA software version 11.1SE (StataCorp, College Station, TX, USA) and GraphPad Prism software version 6 (GraphPad Inc., San Diego, CA, USA).

## 4. Results

We identified 557 patients with non-valvular AF and diabetes who received Edoxaban or VKA treatment. We excluded patients with a follow-up ≤360-days from the first qualifying anticoagulant prescription (*n* = 37) or VKA patients with time in therapeutic range <70% (*n* = 65). Propensity score matching identified 135 Edoxaban (EDO) and 135 VKA recipients who were comparable with respect to demographic and clinical characteristics. INR was not included in matching because it would be inherently higher in the VKA group. The baseline characteristics of the study population before and after propensity score matching are summarized in Table 1. The mean follow-up was 27 ± 3 months. Concerning the EDO group, 117 patients (87%) were on Edoxaban 60 mg once daily (OD and 18 patients (13%) were on Edoxaban 30 mg OD. A total of 8 patients (3 in EDO group vs. 5 in VKA group) experienced thromboembolic events during the follow-up. The incidence rate of TE was 3.0 per 100 person-years; in particular, 1.1 per 100 person-years in EDO group vs. 1.9 per 100 person-years in VKA group (HR: 0.59; 95% CI: 0.14 to 2.52; *p* = 0.48).

**Figure 1:**
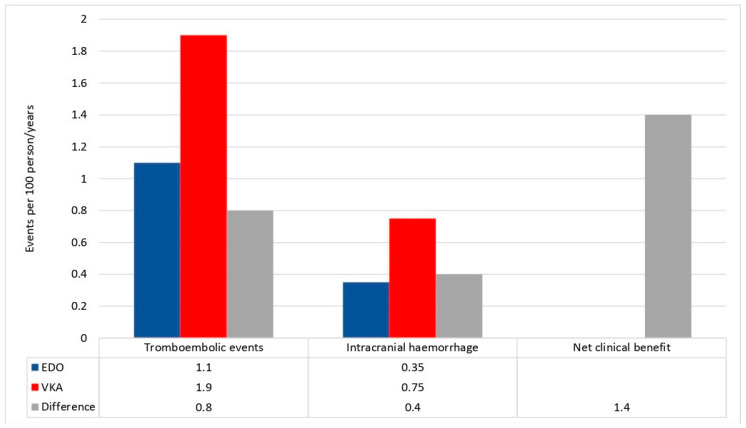
Incidence rates of thromboembolic events and intracranial hemorrhage in Edoxaban (EDO) and vitamin K antagonist (VKA) recipients. Differences (Δ) between incidence rates were used to calculate the net clinical benefit (NCB).

A total of 10 patients (3 in EDO group vs. 7 in VKA group) experienced major bleeding during the follow-up. The incidence rate of MB was 3.7 per 100 person-years; in particular, 1.2 per 100 person-years in EDO group vs. 2.7 per 100 person-years in VKA group (HR: 0.43; 95% CI: 0.10 to 1.40; *p* = 0.14). Among MB, 3 were ICH (1 in EDO group and 2 in VKA group). The incidence rate of ICH was 0.35 per 100 person-years in EDO group vs. 0.74 in VKA group (HR: 0.49; 95% CI: 0.05 to 5.54; *p* = 0.56). Through these incidence rates, we found a positive NCB of Edoxaban over VKAs, equal to +1.39 (Figure 1).

**Figure 2:**
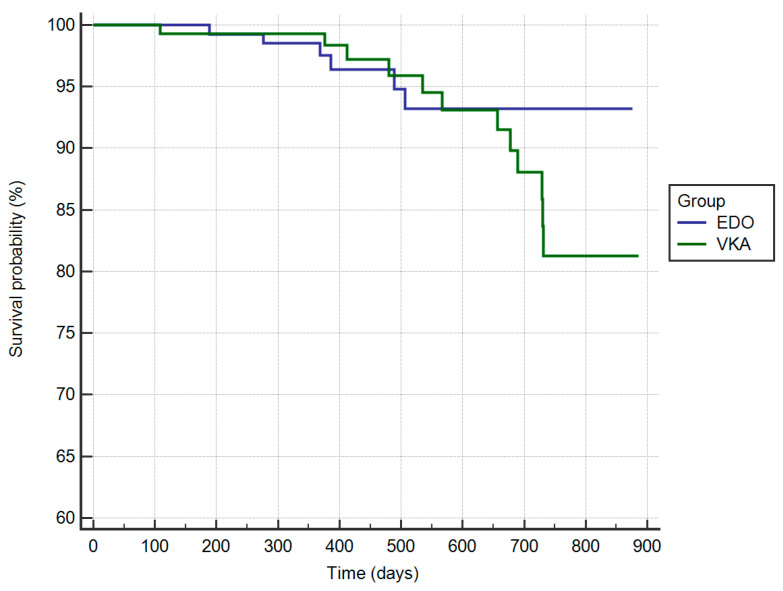
Kaplan-Meier cumulative probability of primary outcome event-free survival Edoxaban (EDO) and vitamin K antagonist (VKA) treatment group.

Figure 2 shows the Kaplan-Meier analysis of survival free from occurrence of primary outcome event, both thromboembolic and hemorrhagic, in the EDO and VKA treatment groups, respectively (HR: 0.65; 95% CI: 0.25 to 1.64; *p* = 0.38).

Two patients died during follow-up (1 in EDO group and 1 in VKA group), both for cancer-related causes. A total of 19 patients reported CRNMB events (8 in EDO group and 11 in the VKA group). The incidence rate of CRNMB was 3.0 per 100 person-years in the EDO group vs. 4.8 in the VKA group (HR: 0.71; 95% CI: 0.28 to 1.82; *p* = 0.48).

The univariate and multivariate results for the variables determining the development of MB and TE are shown in Table 2 and Table 3, respectively. According to this analysis, the presence of insulin therapy (HR: 1.76, *p* = 0.004) was found to be an independent predictor of TE; moreover, the concomitant use of antiplatelet drugs (HR: 2.41, *p* = 0.001) was an independent predictor of MB.

## 5. Discussion

From the subgroup analyses of 4 phase-III trials comparing NOACs with warfarin, AF patients with diabetes were at increased risk of thromboembolic events compared with those without diabetes; the relative safety and efficacy of NOACs versus warfarin were similar, regardless of diabetes status [11,12].

Our results showed a high incidence of primary effectiveness among AF patient with diabetes on oral anticoagulation therapy. As expected, diabetes was associated with an increased risk of thromboembolic events despite oral anticoagulation treatment, probably due to the hyperglycemia-induced endothelial dysfunction [13], and thereby, to increased individual susceptibility to atherosclerosis [14]. Regarding hemorrhagic events, the incidence of MB was relatively low and similar to that reported for the AF patients with diabetes included in NOACs RCT [11,12], supporting the evidence that diabetes doesn’t seem to be a risk predictor of bleeding among AF patients on oral anticoagulation medication [15].

A recent subanalysis of in an ENGAGE AF TIMI 48 (Effective Anticoagulation with Factor Xa Next Generation in Atrial Fibrillation Thrombolysis in Myocardial Infarction 48) trial, including 7624 (36%) patients with history of diabetes mellitus, showed that Edoxaban had similar efficacy compared to warfarin, while reducing bleeding and cardiovascular mortality, irrespective of diabetes [8].

There are few real-world data (RWD) about the clinical performance of NOACs in AF patients with diabetes [16,17], and no RWD are yet available regarding Edoxaban treatment in this clinical setting. The present study investigated the safety and effectiveness of Edoxaban versus VKAs among a propensity score -matching cohort of AF patients with diabetes in a real-world setting. Moreover, we used the net clinical benefit measure to assess the balance of risk and benefit of Edoxaban versus warfarin in a single quantitative measure including the two more fearsome complications of anticoagulant therapy: thromboembolic events and intracranial hemorrhages.

Among our study population, a trend in reduction of both thromboembolic and major bleeding events, including ICH, was observed in AF patients with diabetes on Edoxaban versus VKAs therapy. These results support the hypothesis of a better net clinical benefit of Edoxaban vs. VKAs in AF patients with diabetes, as previously shown in elderly patients [18]; however, our preliminary results need to be confirmed in a larger population.

We found that the presence of insulin therapy and increased levels of glycated hemoglobin were independently associated with the risk of thromboembolic events among our study population. This evidence suggests that diabetic patients on insulin therapy may be more susceptible to AF-related thromboembolism than individuals with less severe forms of diabetes usually not requiring insulin, as shown in the PREFER in AF (European Prevention of Thromboembolic Events—European Registry in Atrial Fibrillation) registry [19]. The pathophysiological mechanisms underlying the possible association between insulin therapy and AF-related thromboembolism in diabetic patients are not completely known. A hypercoagulable state, characterized by a pronounced platelet activation, has been shown in patients with diabetes mellitus [20]; in particular, those with long-lasting disease receiving insulin therapy seem to show a higher tendency to form clots [21].

The presence of insulin therapy may be considered per se a marker of advanced stages of disease, which are more likely to be characterized by hyperglycemia and endothelial cell dysfunction [22]. Moreover, chronic exposure to elevated levels of advanced glycosylation end-products and the direct effect exogenous insulin, determining increased serum levels of insulin in cases of insulin resistance, might be responsible for vascular injury and premature atherosclerosis [23]. Our data confirmed the association between glycemic status, as reflected by glycated hemoglobin serum levels, and thromboembolic risk in patients with atrial fibrillation and diabetes mellitus [24].

The concomitant use of antiplatelet drugs was found to be the only independent predictor of major bleedings in AF patients with diabetes on oral anticoagulation therapy, confirming that the combination of antiplatelet drugs with oral anticoagulants increases the risk of bleeding [25,26].

The small sample size, relatively short follow-up time and low number of endpoint events were major limitations in the present study; however, to date, this is the first real-world evidence focusing on the use of Edoxaban versus VKAs in a cohort of AF patients with diabetes. VKA patients with time in therapeutic range <70% were a priori excluded from the analysis, because we were seeking to compare Edoxaban to optimal VKA therapy. Moreover, AF patients treated with a not recommended Edoxaban dosage (either under- or over-treated) were excluded from the analysis by the PSM. Notably, only a few AF patients (2.6%) received an off-label dose of Edoxaban; this might be related, on the one hand, to increasing awareness about the clinical consequences of treating patients with not recommended dosages, and on the other hand, to clear criteria for Edoxaban dosing adjustment.

## 6. Conclusions

There are few real-world data on the clinical profiles of direct oral anticoagulants among AF patients with diabetes. Our study supports the hypothesis of a favorable net clinical benefit of Edoxaban over VKA in this population. Further prospective studies including a large number of patients are necessary to confirm our preliminary observations.

## Figures and Tables

**Table 1:** Baseline characteristics of the study population before and after propensity matching.

Variable	Before Propensity Score Matching			After Propensity Score Matching		
	EDO (*n* = 230)	VKA (*n* = 327)	*p*-Value	EDO (*n* = 135)	VKA (*n* = 135)	*p*-Value
Age (years)	69.1 ± 9.1	76.9 ± 1.4	<0.001	69.2 ± 5.1	70.3 ± 5.0	0.72
Female (*n*, %)	105 (45.5)	141 (43.1)	0.59	56 (41.4)	55 (40.7)	0.71
BMI (kg/m^2^)	24.8 ± 4.5	28.8 ± 4.1	0.08	27.3 ± 3.3	27.6 ± 3.3	0.77
Paroxysmal AF (*n*, %)	57 (25)	88 (27)	0.78	34 (25)	35 (26)	0.78
Persistent AF (*n*, %)	80 (35)	121 (37)	0.89	46 (34)	49 (36)	0.81
Permanent AF (*n*, %)	92 (40)	118 (36)	0.82	55 (41)	51 (38)	0.81
Hypertension (*n*, %)	111 (48.5)	197 (60.2)	0.001	72 (53.1)	75 (55.6)	0.56
Heart failure (*n*, %)	39 (16.8)	92 (28.2)	0.001	28 (20.8)	29 (21.4)	0.7
Prior stroke/TIA (*n*, %)	64 (27.8)	116 (35.4)	0.001	39 (28.8)	40 (29.4)	0.7
Prior major bleeding (*n*, %)	10 (4.3)	24 (7.5)	0.06	5 (3.5)	5 (3.5)	0.7
Prior MI (*n*, %)	19 (8.3)	43 (13.2)	0.02	8 (5.7)	9 (6.5)	0.7
CKD (*n*, %)	36 (15.5)	53 (16.1)	0.57	21 (15.4)	22 (16.1)	0.57
CHA2DS2VASc score	4.2 ± 1.7	4.6 ± 1.6	0.57	4.3 ± 1.4	4.5 ± 1.5	0.56
HAS-BLED score	3.4 ± 1.4	3.6 ± 1.3	0.57	3.5 ± 1.1	3.6 ± 1.7	0.56
LVEF (%)	49.5 ± 6.4	40.3 ± 7.1	0.003	44.4 ± 5.2	42.3 ± 4.8	0.8
Glycated hemoglobin (%)	8.9 ± 1.1	9.6 ± 2.3	0.89	8.2 ± 1.5	8.9 ± 1.6	0.78
Insulin Therapy (*n*, %)	55 (24)	114 (35)	0.002	27 (20)	27 (20)	0.8
Antiplatelets (*n*, %)	44 (19)	39 (12)	0.05	15 (11)	15 (11)	0.8
Oral hypoglycemic agents (*n*, %)	182 (79)	265 (81)	0.57	108 (80)	109 (81)	0.9
Lipid lowering drugs (*n*, %)	182 (79)	262 (80)	0.57	128 (94.8)	128 (94.8)	0.9
Edoxaban 60 mg (*n*, %)	200 (87)	−		117 (87)		
Edoxaban 30 mg (*n*, %)	30 (13)	−		18 (13)		
Acenocumarolo (*n*, %)		42 (13)			2 (1.5)	
Warfarin (*n*, %)	−	284 (87)			133 (98.5)	
Therapeutic Dosage (*n*, %)	216 (94)			100		
Underdosing (*n*, %)	11 (5)			−		
Overdosing (*n*, %)	2 (1)			−		

Mean ± SD unless otherwise stated. BMI: body mass index; GFR: glomerular filtration rate; DOAC: direct oral anticoagulants; LAVI: left atrial volume index; MI: myocardial infarction; SD, standard deviation; TIA, transient ischemic attack, VKA, Vitamin K antagonists, LVEF: left ventricle ejection fraction.

**Table 2:** Univariate and multivariate analysis results for variables determining the development of major bleeding events.

Predictors of Major Bleeding Events		
Variable	HR (95% CI; Univariable)	HR (95% CI: Multivariable)
Age	1.10 (0.87–1.12, *p* = 0.965)	−
Female sex	0.99 (0.71–1.38, *p* = 0.969)	−
BMI	1.00 (0.98–1.02, *p* = 0.973)	−
Hypertension	0.69 (0.43–1.12, *p* = 0.132)	−
CAD	0.70 (0.43–1.12, *p* = 0.134)	−
Heart failure	1.47 (0.60–3.59, *p* = 0.396)	−
CKD	0.89 (0.29–2.67, *p* = 0.832)	−
Prior Stroke/TIA	1.05 (0.71–1.55, *p* = 0.796)	−
Past Bleeding	1.08 (0.86–1.35, *p* = 0.515)	−
Glycated Hemoglobin	1.24 (0.48–3.61, *p* = 0.396)	−
Oral Hypoglycemic Agents	1.38 (1.10–1.78, *p* = 0.059)	NS
Antiplatelet Drug	2.54 (1.53–4.22, *p* < 0.001)	2.41 (1.43–4.07, *p* = 0.001)
Insulin Therapy	1.76 (1.20–2.59, *p* = 0.004)	NS

HR: hazard ratio; CI: confidence interval; BMI: body mass index; DM: diabetes mellitus; CAD: coronary artery disease; CKD: chronic kidney disease; TIA: transient ischemic attack; NS: non significative.

**Table 3:** Univariate and multivariate analysis results for variables determining the development of thromboembolic events.

Predictors of Thromboembolic Events		
Variable	HR (95% CI; Univariable)	HR (95% CI; Multivariable)
Age	1.23 (0.46–3.29, *p* = 0.020)	NS
Female sex	1.14 (1.02–1.27, *p* = 0.687)	−
BMI	0.86 (0.32–2.31, *p* = 0.764)	−
Hypertension	0.94 (0.74–1.19, *p* = 0.599)	−
CAD	1.26 (0.60–2.68, *p* = 0.540)	−
Heart failure	1.35 (0.97–1.86, *p* = 0.071)	NS
CKD	1.14 (0.83–1.56, *p* = 0.413)	−
Prior stroke/TIA	1.28 (1.00–1.64, *p* = 0.061)	NS
Past bleeding	1.14 (0.83–1.56, *p* = 0.413)	−
Glycated hemoglobin	1.15 (1.04–1.27, *p* = 0.005)	1.17 (1.06–1.29, *p* = 0.002)
Oral hypoglycemic agents	1.28 (0.65–2.56, *p* = 0.440)	−
Antiplatelet drug (%)	1.23 (0.46–3.29, *p* = 0.687)	−
Insulin therapy (%)	1.65 (1.23–2.22, *p* = 0.003)	1.76 (1.20–2.59, *p* = 0.004)

HR: hazard ratio; CI: confidence interval; BMI: body mass index; DM: diabetes mellitus; CAD: coronary artery disease; CKD: chronic kidney disease; TIA: transient ischemic attack; NS: non significative.
